# Rapid microarray-based assay for detection of pyrazinamide resistant *Mycobacterium tuberculosis*

**DOI:** 10.1016/j.diagmicrobio.2018.12.011

**Published:** 2019-06

**Authors:** Juliane Havlicek, Beatrice Dachsel, Peter Slickers, Sönke Andres, Patrick Beckert, Silke Feuerriegel, Stefan Niemann, Matthias Merker, Ines Labugger

**Affiliations:** a Alere Technologies GmbH, Jena, Germany; b National Reference Center for Mycobacteria, Research Center Borstel, Borstel, Germany; c Molecular and Experimental Mycobacteriology, Research Center Borstel, Germany; d German Center for Infection Research, Partner site Hamburg-Lübeck-, Borstel, -Riems, Germany

**Keywords:** *Mycobacterium tuberculosis*, Pyrazinamide resistance, Microarray

## Abstract

Pyrazinamide (PZA) is a key antibiotic for the treatment of drug susceptible tuberculosis. PZA-resistance is mainly mediated by mutations in the *pncA* gene; however the current gold standard is a phenotypic drug susceptibility test requiring a well-adjusted pH-value for reliable results.

Our melting curve assay detects a non-wild type genotype in selected *pncA* regions in at least 3750 gene copies/mL within 2.5 hours. The prototype assay was further evaluated by analyzing 271 *Mycobacterium tuberculosis* complex isolates from Swaziland originating from a previously published drug resistance survey and including 118 isolates with *pncA* mutations. Sensitivity was 83% (95% CI 75–89%) and specificity was 100% (95% CI 98–100%). Under consideration of further improvements with regard to the target range our melting curve assay has the potential as a rapid rule-in test for PZA susceptibility (wild type *pncA*), however false resistant results (mutant *pncA*, but PZA susceptible) cannot be ruled out completely.

## Highlights

• Cartridge-based automated detection of *M. tuberculosis* complex *pncA* mutants
• High specificity could allow for the prediction of PZA susceptibility
• Works with heat inactivated culture material
• Target range to be improved and applicability on direct patient samples to be shown

## 1 Introduction

Tuberculosis (TB), caused by *Mycobacterium tuberculosis* complex (MTBC) strains, remains one of the most frequent infectious diseases worldwide (WHO, 2017). Pyrazinamide (PZA) is a key drug for TB treatment with a very high sterilizing activity and thus it is able to accelerate the elimination of persistent bacteria in conjunction with other antibiotics. A major advantage is its potential to shorten the duration of drug susceptible TB treatment to 6 months (Caminero et al., 2012). In addition, resistance towards PZA is widely spread among multidrug resistant (MDR, isoniazid and rifampicin resistant) MTBC isolates with rates between 38% to 60.5% depending on the world region (Chiu et al., 2011, Daneau et al., 2016, Jonmalung et al., 2010, Kurbatova et al., 2013, Mphahlele et al., 2008, Pierre-Audigier et al., 2012, Whitfield et al., 2015a, Zignol et al., 2016). Due to its high frequency and the increased risk of co-resistance to other drugs, rapid detection of PZA resistance is essential to control the MDR-TB epidemic (Whitfield et al., 2015a).

However, PZA resistance testing is not routinely performed (WHO, 2016). Culture-based methods are still the gold standard for diagnosis and resistance detection in MTBC strains (Smith, 2003). The BACTEC MGIT 960 (BD, Franklin Lakes, NJ, USA) system is based on an oxygen sensor which is embedded in silicon at the bottom of a modified Middlebrook broth tube. Mycobacterial growth is detected by fluorescence due to the oxygen reduction (Tortoli et al., 1999). However, the testing of PZA resistance is most robust under acidic conditions (pH 5.5 to 6.0), which also inhibit the growth of mycobacteria, leading to low reproducibility, controversial test results, and thus makes culture not the appropriate method to detect PZA resistance. Additionally, phenotypic resistance testing is limited due to the long turn-around time (Cui et al., 2013, Morlock et al., 2000, Zimic et al., 2012). For this reason molecular test methods become even more important. But targeted molecular assays are challenged by diverse mutations scattered across the *pncA* gene (561 bp plus promotor region) that is coding for the pyrazinamidase PncA, the drug activator (Miotto et al., 2014, Palomino and Martin, 2014). DNA sequencing approaches like whole genome sequencing (WGS) or amplicon sequencing can overcome this limitation (Park et al., 2018, Walker et al., 2017). Nevertheless, sequencing is expensive, technically demanding and yet not suitable as a point of care test (Willby et al., 2017). Further, different mutations likely alter the enzymatic activity of the drug activator PncA only gradually. Thus, recent approaches sought to assess the clinical relevance of hundreds of mutations but yet not with a common unambiguous high confident catalogue (Miotto et al., 2017, Walker et al., 2017, Yadon et al., 2017).

Another concept is to rule-in PZA susceptibility (instead of resistance) that is based on the presence of a *pncA* wild type sequence and was developed by Driesen et al. using a line probe assay (LPA) that employs 48 wild type probes to cover the entire *pncA* gene and the putative promotor region up to nucleotide −18 (Driesen et al., 2018).

Here, we aimed to develop a rapid point-of-care assay with a reduced risk of DNA cross contaminations, e.g. which might occur during PCR steps for LPA or WGS, and interrogating larger parts of the *pncA* gene. Our assay is a closed cartridge system and based on a melting curve technology recently published for the detection of mutations, which are associated with a Rifampicin-, Isoniazid- and Fluoroquinolone resistance (Havlicek et al., 2018). For the PZA-assay, a non-wild type sequence is detected by a shift of the melting curve of the affected probe(s). We present an evaluation of our prototype assay including the determination of the detection limit, the characterization of the assay performance with clinical isolates from Swaziland as well as with heat inactivated culture material in comparison to genomic DNA.

## 2 Materials and methods

### 2.1 Strains and culture material

The MTBC strains for the development and the clinical test evaluation (Table S1) have been cultivated according to standard routine conditions at the German National Reference Research Center, Borstel, Germany. For inactivation the cell culture material of each strain was treated with ultrasound and heated at 99 °C for 15 min. Heat inactivated culture material was also tested with our assay and is in the following referred to as cell lysates.

In total, 271 clinical MTBC isolates, which were derived from a national survey of drug resistance in Swaziland (Sanchez-Padilla et al., 2012), were used for the clinical evaluation of the melting curve assay. All isolates were previously analyzed by Sanger sequencing of the *pncA* gene using flanking primers from position −104 to 560 out of 561 coding nucleotides. The sequence analysis showed wild type genotype for 153 isolates and different mutations in the analyzed *pncA* sequence for 118 isolates.

### 2.2 Preparation of genomic DNA and synthetic plasmids

The method for isolation of genomic DNA was described in detail by Havlicek et al. (2018). To test rare mutations (Ala25Ala, Ser74Ser, Leu116Arg and Asn147Asn) plasmids with the respective sequences were generated by Eurofins Genomics GmbH, Ebersberg, Germany.

### 2.3 Primer, TaqMan® probes and array probes

Sequence information for all primer, array probes and TaqMan® probes are available in Table S2. The array probes had a C7-amino linker for immobilization on the solid array phase. The forward primers for the melting curve analysis were Cy5 labeled at the 5′ end, whereas the reverse primers for the melting curve analysis had no label as well as the primers for the TaqMan® analysis. All oligonucleotides were synthesized by Eurogentec, Cologne, Germany. The description, how the efficiency of the target amplification was determined, is available in the supplemental material.

### 2.4 Melting curve assay

**Fig. 1:**
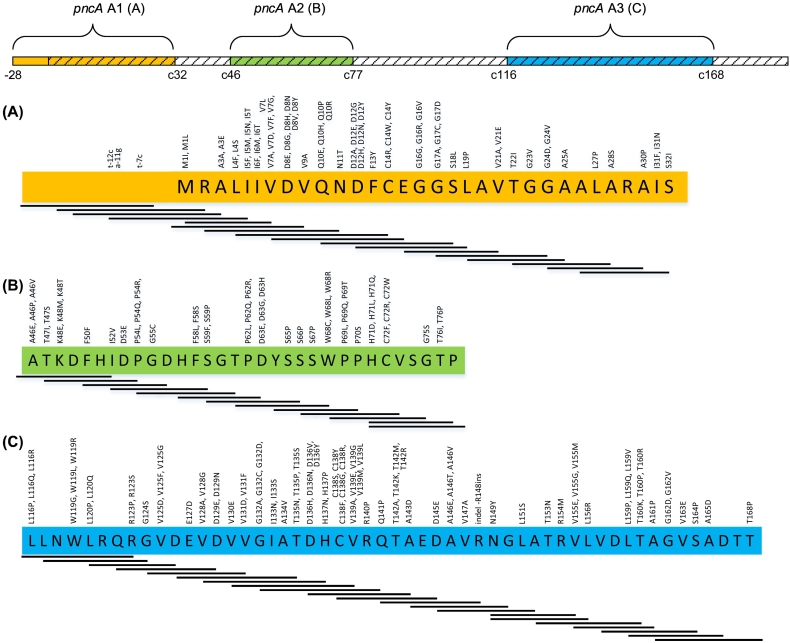
Splitting of the *pncA* gene in three amplicons. Our melting curve assay covers three gene regions: nucleotide −28 to codon 32 (yellow), codon 46 to codon 77 (green) and codon 116 to codon 168 (blue). The shape with the diagonal lines represents the whole *pncA* gene. In the detailed presentations (A, B and C) the amino acid sequence with one-letter code is given. Below, the wild type probes for the detection of the respective regions are displayed. High-confidence mutation in the *pncA* gene detected by Miotto et al. (2017) and PZA resistance associated mutations detected by Yadon et al. (2017) are listed above, respectively.

**Fig. 2:**
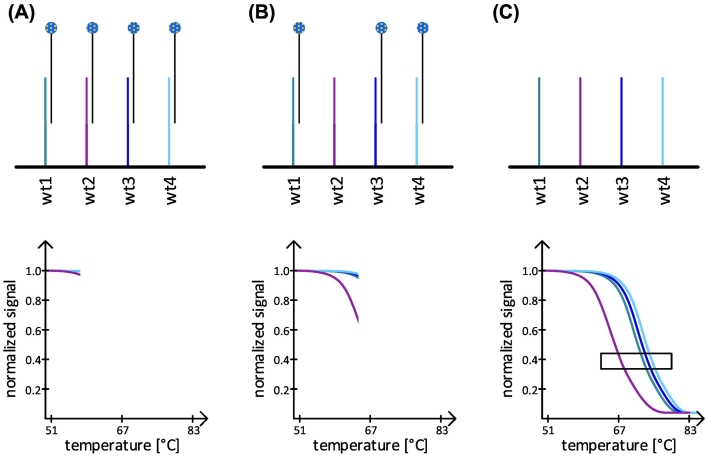
Detection principle of the melting curve assay. Cy5 labeled amplicons hybridized with immobilized probes on the array which represent the wild type (wt) genotype (A). With increasing temperature all amplicon-probe bindings, which carried a mismatch due to a *pncA* mutation in the analyzed sample, were dissociate first (B, purple line). With a further increase in temperature *pncA* wild type amplicons dissociate also from their matching probe (C, blue lines). The rectangle indicates the area which was used to differentiate between a wild type and non-wild type *pncA* genotype. In case of a *pncA* mutation the melting curve shows a significant shift to lower temperatures compared to wild type curve.

Initially a multiplex amplification of the three specific amplicons (*pncA* A1, *pncA* A2 and *pncA* A3) was performed to cover the defined gene regions: nucleotide −28 to codon 32, codon 46 to codon 77 and codon 116 to codon 168 (Fig. 1). Cy5 labeled forward primers were used to obtain fluorescence-labeled amplicons. In a next step the amplicons were denatured, hybridized on 51 overlapping array probes which represent the wild type *pncA* genotype and were immobilized on the solid phase (Fig. 2, A). After a washing step a melting step was performed in a temperature range from 51 °C to 83 °C. With increasing temperature amplicon-probe bindings were dissociated (Fig. 2, B). If the sample was *pncA* wild type genotype, the generated amplicons were complementary to the spotted probes and dissociated at a higher temperature (Fig. 2, C). In addition to the *pncA* wild type probes, three probes specific for the identification of the phylogenetic mutations of *M. canettii* (Ala46Ala (GC(G/A)); Somoskovi et al., 2007), *M. bovis* (His57Asp ((C/G)AC); Scorpio et al., 1997) or the CAS lineage of *M. tuberculosis* (Ser65Ser (TC(T/G)); Stavrum et al., 2009) and a probe for the silent mutation Ser74Ser (AG(C/T)) were present on the array. Further, internal controls were implemented, i.e. a negative hybridization control, an artificial amplification control and a positive hybridization control.

The amplification and melting curve assay was performed in a prototype cartridge (Alere Technologies GmbH, Jena, Germany) with a reaction volume of 100 μL comprising 75 mM TRIS hydrochloride pH 8.5 (Alere Technologies GmbH, Jena, Germany), 3 mM magnesium chloride (Sigma-Aldrich, St. Louis, USA), 1 betaine tablet (resulting in 2 M betaine, University Friedrich-Alexander, Erlangen, Germany), 100 mM tetramethylammonium chloride (Alere Technologies GmbH, Jena, Germany), 0.2 mM deoxynucleoside triphosphate mixture (Thermo Fisher Scientific, Waltham, USA), 0.6 μM of each Cy5 labeled forward primer (Table S2), 0.2 μM of each reverse primer (Table S2), 25 U of BTR hotstart *taq* (Biotech rabbit, Henningsdorf, Germany), 10^4^ copies per reaction of the artificial process control (Eurofins Genomics GmbH, Ebersberg, Germany) and 1 μL of genomic *M. tuberculosis* DNA or cell lysates. Prior to test all used genomic DNAs and the clinical DNA isolates were diluted 1:50,000 and cell lysates were diluted 1:100 in TRIS-EDTA buffer (Sigma-Aldrich, St. Louis, USA). Negative controls were run without any template in the same reaction mix. The amplification was performed at 95 °C for 2 min following 50 cycles at 95 °C for 10 s, 64 °C for 30 s and 72 °C for 30 s. After amplification a magnesium pellet (resulting in 200 mM magnesium chloride, Alere Technologies GmbH, Jena, Germany) was dissolved in the reaction mix. Amplicons were denatured at 95 °C for 2 min and hybridized on array probes at 30 °C for 2 min. An array wash step was performed using buffer WB2.10 (Alere Technologies GmbH, Jena, Germany) which was also rised to 200 mM magnesium chloride by solving a magnesium chloride pellet. Finally, the dissociation of labeled strands from the spots on the array was monitored by raising the temperature stepwise from 51 °C to 83 °C with 2 °C increments and acquiring an image at the end of each temperature step.

### 2.5 Data processing and analysis

The acquired image series was analyzed using the Iconoclust software (Alere Technologies GmbH, Jena, Germany). The array spots were identified using a defined grid. After segmentation, spot pixels and backgrounds were sorted according to their brightness and the respective values were determined. The background was subtracted from the brightness value to determine the raw signal for each spot. A continuous signal for each probe was received by performing an interpolation. If the raw signal exceeded a fluorescence level of 0.01, the signal was normalized. Due to similar melting behavior 51 wild type probes were categorized into six evaluation groups (Table S2). The non-wild type detection was based on a shift of the melting curve of the affected probe compared to the other probes in one evaluation group. This shift could be proven by an outlier testing of the determined melting temperatures at a defined signal level (Fig. 2, C). For outlier testing, we applied a one-sided Tietjen-Moore test. The Tietjen-Moore test is appropriate to detect multiple outliers in a univariate data set that follows a relatively normal distribution (Tietjen and Moore, 1972). If the Tietjen-Moore test detected an outlier within one evaluation group, a mutation was present in the DNA sequence of the tested sample.

### 2.6 Sensitivity and specificity

Sanger sequencing data of a previously published MTBC collection from a country wide drug resistance survey in Swaziland and selected reference isolates were considered as gold standard for the identification of mutations in the *pncA* gene. Confidence intervals for Sensitivity and Specificity were calculated using the exact Clopper-Pearson method.

## 3 Results

### 3.1 Detection limit

**Fig. 3:**
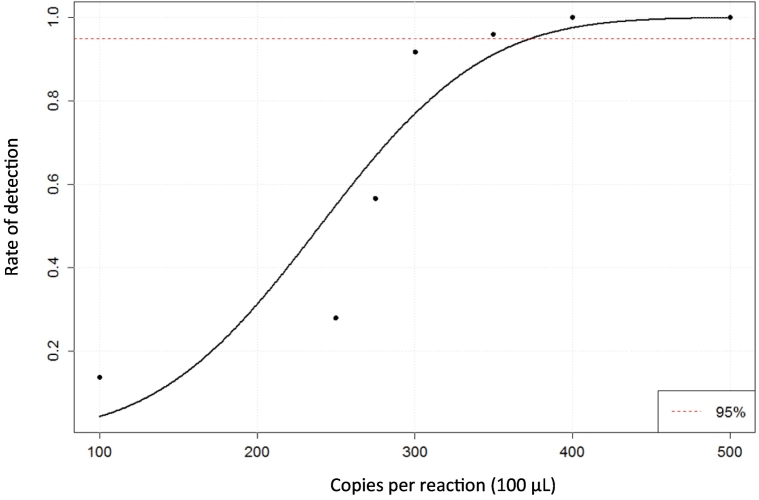
Detection limit of the melting curve assay. Multiplex amplification of the three target regions *pncA* A1, *pncA* A2 and *pncA* A3 was performed using the prototype cartridge and the Alere™ q analyzer. The detection limit was determined to be 3750 copies/mL with a 95% confidence interval of 3440 copies/mL to 4250 copies/mL, 25 tests were performed. The dashed line shows the threshold where in 95% of cases MTBC DNA could be detected.

The detection limit of our melting curve assay was determined using purified genomic DNA from *M. tuberculosis* reference strain H37Rv in a concentration range of 1000 to 5000 copies/mL. An increase of the detection rate was observed with rising DNA concentrations. The detection limit of the melting curve assay was 3750 copies/mL (95% confidence interval 3440–4250 copies/mL) (Fig. 3).

### 3.2 Detection of non-wild-type genotypes

**Fig. 4:**
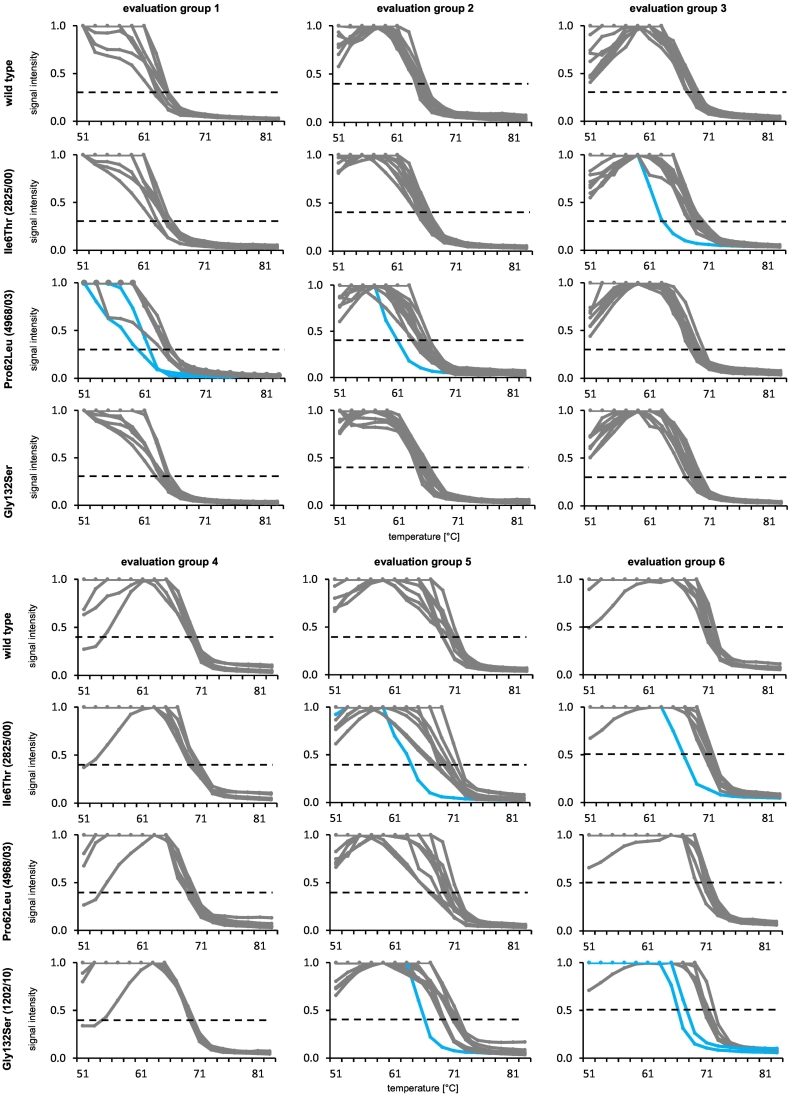
Melting curves generated with genomic DNAs representing different *pncA* genotypes are shown for each of the six evaluation groups. Each curve represents one probe, the composition of each evaluation group can be found in Table S2. The y-axis shows the signal intensity of the melting curve depending on the temperature (x-axis). The defined signal threshold for each evaluation group is represented by a dashed line. The gray colored curves represented in each case the melting curve of a probe if a *pncA* wild type genotype was investigated. All gray colored curves showed a similar behavior, thus no outlier was detected according to the defined evaluation algorithm. Contrary, if a tested isolate carried a mutation, the affected probe(s) showed an altered melting behavior in comparison to other probes of the respective evaluation group; these outliers were presented as blue colored curves. Melting curves generated with genomic DNAs representing different *pncA* genotypes are shown for each of the six evaluation groups. Each curve represents one probe, the composition of each evaluation group can be found in Table S2. The y-axis shows the signal intensity of the melting curve depending on the temperature (x-axis). The defined signal threshold for each evaluation group is represented by a dashed line. The gray colored curves represented in each case the melting curve of a probe if a *pncA* wild type genotype was investigated. All gray colored curves showed a similar behavior, thus no outlier was detected according to the defined evaluation algorithm. Contrary, if a tested isolate carried a mutation, the affected probe(s) showed an altered melting behavior in comparison to other probes of the respective evaluation group; these outliers were presented as blue colored curves.

To characterize the performance of our melting curve assay, a set of 36 different sequence-determined *M. tuberculosis* isolates and four plasmids with different *pncA* mutations were tested. Seven (7) of these isolates and plasmids belong to amplicon *pncA* A1, 15 to amplicon *pncA* A2 and 18 to amplicon *pncA* A3 (Table S1). The melting curves of the wild type and three different mutations (Ile6Thr, Pro62Leu and Gly132Ser) are exemplary given in Fig. 4. The results for all tested isolates and plasmids are available in Table S3. In case of wild type genotype all probes in an evaluation group showed the same melting behavior. No shift of any curve and consequently no outlier was detected. All mutations could be detected due to at least one outlier in an evaluation group.

The mutation Ile31Ser in isolate 6103/09 was verified by a manual evaluation as the shift of the affected probe was not clear enough to get automatically detected by the software. The following isolates (and mutations) showed additional outlier signals, beside the signal induced by the corresponding mutant probe: 7683/04 (Ser59Pro), 4897/05 (Tyr64Asp), 1879/10 (Ser67Pro), 10,735/04 (Ile133Thr), as well as the plasmid carrying the mutation Asn147Asn. The phylogenetic SNPs of *M. canettii*, *M. bovis* and *M.tuberculosis* Delhi/CAS lineage as well as the silent mutation at position 74 in the *pncA* gene were correctly identified in the respective isolates or plasmids.

### 3.3 Clinical isolates

The performance of our melting curve assay was evaluated with 271 clinical isolates from Swaziland (Table S1). With the melting curve assay we have not detected any mutations in 153 Sanger sequencing confirmed *pncA* wild type isolates. Among 118 isolates with 21 different *pncA* mutations, 98 (83.05%) were detected as a non-wild type genotype (Table 1). Four (4) of these clinical isolates belong to amplicon *pncA* A1, 45 to amplicon *pncA* A2 and 49 to amplicon *pncA* A3. The most common mutations were Arg154Gly (26.5%), His51Asp (14.8%), Leu151Ser (6.1%), and a deletion from codon 125 to 129 (6.1%). In two isolates the mutation Ser65Ser could be identified which is specific for the Delhi/CAS lineage. In 20 cases, the isolates carried *pncA* mutations outside the detection range of our melting curve assay. In comparison to Sanger sequencing, the melting curve assay showed a sensitivity of 83.05% (95% CI 75.04% to 89.33%) and a specificity of 100% (95% CI 97.62% to 100.00%).

To assess the performance of our current assay for MTBC strains from other geographical regions, we did an ‘in silico’ analysis for 1000 samples from Samara, Russia with available genome sequencing data (Casali et al., 2014). Assuming again only a limitation based on the target range, our assay could reach a sensitivity of 74.4% in the Russian cohort.

**Table 1:** Clinical evaluation of *M. tuberculosis* isolates from Swaziland.

	*pncA* sanger sequencing		Sensitivity [%] (95% CI)	Specificity [%] (95% CI)
	Wild-type (n)	Mutant (n)		
Melting curve assay – positive	0	98	83.05 (75.04 to 89.33)	100 (97.62 to 100.00)
Melting curve assay – negative	153	20		
**Total**	**153**	**118**		

### 3.4 Assay performance using cell lysates

The potential of our melting curve assay for use in a non-specialized laboratory environment was assessed with cell lysates, i.e. heat inactivated culture material. For this purpose, six different *M. tuberculosis* cell lysates, one with *pncA* wild type genotype and five with different *pncA* mutations (His51Asp, Del125–129, Asp136Ala, Leu151Ser, and Arg154Gly), were analyzed with this assay. Results were compared to purified genomic DNA. All lysates with mutations could be identified as a non-wild type genotype and no difference in the assay performance between genomic DNA and cell lysates was observed (Table S4).

## 4 Discussion

We could show the potential of our cartridge based melting curve assay to predict PZA susceptibility, i.e. as inferred from a *pncA* wild type genotype, with a specificity of 100%. The main limitation of this prototype assay is the restricted target range, yet only covering three amplicon regions: −28 to codon 32 (*pncA* A1), codon 46 to codon 77 (*pncA* A2) and codon 116 to codon 168 (*pncA* A3), and the risk of detecting currently unknown phylogenetic or other unique non-resistance related mutations as false positives. Further, the performance on direct clinical specimen, i.e. sputum, needs to be demonstrated. The main advantage, compared to available LPAs, and next generation sequencing approaches such as whole genome or amplicon sequencing is the closed cartridge system that reduces contamination risks and allows for the application as a rapid point-of-care test in resource limited settings.

Currently the gold standard for resistance detection is culture-based drug susceptibility testing using BACTEC MGIT 960 PZA medium (Whitfield et al., 2015b). Nevertheless, false resistance results have been reported with this assay, which is caused by an alkalization of the medium due to a high inoculum size (Heifets, 2002) or the presence of bovine serum albumin (Zhang et al., 2002). Another limitation is the long turnaround time due to the need of at least a primary culture, often PZA DST is indeed performed with a secondary culture (Siddiqi et al., 2012). A direct inoculation of PZA containing MGIT tubes with sputum specimens is more prone to invalid results due to possible contaminations, insufficient growth and/or too small inoculums (Libonati et al., 1988, Siddiqi et al., 2012).

To overcome the limitations of phenotypic tests, molecular tests have been developed. Recently, NGS-based resistance diagnostic has undergone a paradigm change by demonstrating its applicability in a routine reference laboratory setting in the UK and thus allowing to fade out phenotypic DSTs for first-line drugs, including PZA (CRyPTIC Consortium, 2018). But still, NGS approaches and setup/maintenance of related bioinformatics pipelines are cost intensive and require complex laboratory equipment which currently hampers its use in resource-limited settings (Pankhust et al., 2016).

Especially high incidence settings in Eastern Europe and Africa might benefit more in the upcoming years from robust automated test systems that generate a rapid interpretation of DST results from molecular data. On these lines, available PZA-LPAs have shown a high potential for false-positive results due to the use of nested PCR (Ando et al., 2010, Sekiguchi et al., 2007) or problems with the test interpretation due to the highly variable colorization of the different probes and a reliable detection of deletion and insertions (Willby et al., 2017).

Our prototype melting curve assay showed high specificity for the detection of a *pncA* wild type sequence in the investigated target regions, uses a closed cartridge system to reduce contamination risks and has a turnaround time of 2.5 hours with an automated evaluation of the results. Although the concept of inferring PZA susceptibility from a *pncA* wild type sequence bears the risk of false resistant (mutant *pncA*, but PZA susceptible) results, it would allow clinicians for a well-informed inclusion of PZA into early MDR-TB regimens. For the MTBC collection from Swaziland there were just 2/118 (1.7%) *pncA* mutant isolates (*pncA* L19P, and L151S) which tested phenotypically susceptible. Vice versa, there was only 1/113 (0.9%) phenotypically resistant isolates with a *pncA* wild type sequence which would have been misclassified as PZA-susceptible and might harbor another resistance mechanism. Of note, fully first-line susceptible isolates (with a *pncA* wild type sequence) were not tested phenotypically during the drug resistance survey.

Especially high PZA resistance rates (>50%) among MDR-TB patients in Eastern Europe (Balabanova et al., 2017, Lange et al., 2016) makes a solid and conservative prediction of PZA susceptibility valuable and could be a corner stone for an early individualized diagnostic algorithm.

Few adjustments were made for the automatic evaluation. This includes four isolates (carrying the mutations Ser59Pro, Tyr64Asp, Ser67Pro, and Ile133Thr) that showed another outlier signal (not related to the actual mutation) due to the low scattering behavior of other probes in the respective evaluation group, for which we adapted the evaluation routine. Furthermore, in one isolate the mutation Ile31Ser, located at the end of amplicon *pncA* A1, was difficult to detect and needs to be improved within future design amendments. In addition four mutation probes for the detection of *M. canettii*, *M. bovis*, the *M. tuberculosis* lineage Delhi/CAS and a silent mutation at position 74 were added to the array. The mutation probe Ala46Ala is specific for the *M. canettii* species and can be used as phylogenetic marker to distinguish MTBC isolates (Goh et al., 2001, Pfyffer et al., 1998, Somoskovi et al., 2007, van Soolingen et al., 1997). *M. bovis* strains (also part of the MTBC) can be identified by the mutation His57Asp which also leads to PZA resistance (Scorpio et al., 1997, Scorpio and Zhang, 1996). The third mutation Ser65Ser is a marker for the *M. tuberculosis* lineage Delhi/CAS and not related to PZA resistance (Juréen et al., 2008, Stavrum et al., 2009). The probe for detection of the silent mutation Ser74Ser was implemented to exclude false-positive test results. We cannot completely rule out putative artifacts originating from the presence of non-tuberculous mycobacterial DNA in patient specimen.

However, based on routine MTBC culture material we overserved a high specificity of 100% analyzing 271 clinical isolates from a recent drug resistance survey from Swaziland, considering Sanger sequencing results of the *pncA* gene as gold standard for the presence/absence of mutations. In total, 153 of the tested isolates were *pncA* wild type and showed an appropriate behavior in our melting curve assay. The remaining 118 isolates carried different mutations of which 98 could be identified as a non-wild type genotype. The following mutations in 20 isolates could not be detected: Leu35Arg, Lys96Arg, Gly97Cys, Gly97Asp, Tyr103Stop, Thr114Met, and Met175Thr. These mutations were located outside of defined *pncA* amplicon regions resulting in a moderate sensitivity of 83% for the Swaziland dataset and 74% in an in silico analysis for the Samara dataset.

## 5 Conclusion

The current prototype cartridge comprises 57 different probes but has a maximum capacity of 88 probes to be analyzed by microarray hybridization. We envisage to cover the entire *pncA* gene (including promotor region) which would allow to rule-in PZA susceptibility inferred from a *pncA* wild type sequence in clinical isolates with a high specificity. The assay already detects reliably MTBC DNA down to 3750 gene copies/mL with a turnaround time of 2.5 hours and works with genomic DNA as well as with cell lysates. The test can be automatically performed in a closed cartridge using the battery powered Alere™ q analyzer and has the potential to be applied in resource-limited settings as a point-of-care test allowing for a rapid inclusion of PZA into early treatment regimens.

The following are the supplementary data related to this article.

Table S1 Information regarding investigated isolates. Table S1

Table S2 Primers, probes and TaqMan® probes. Table S2

Table S3 Test results for the analysis of different *pncA* isolates using the melting curve assay. Table S3

Table S4 Results of corresponding pairs of genomic DNA and crude culture material from *pncA* isolates in the melting curve assay. Table S4

Supplementary material Image 1
