# Supplementary material for: Rapid microarray-based assay for detection of pyrazinamide resistant Mycobacterium tuberculosis
Source: Diagn Microbiol Infect Dis. 2019 Jun;94(2):147–54. doi: 10.1016/j.diagmicrobio.2018.12.011 (PMC6531379; doi:10.1016/j.diagmicrobio.2018.12.011)
Supplement: Table S1 — Information regarding investigated isolates. [file mmc1.docx]

Table S1. Information regarding investigated isolates.

Analyzed M. tuberculosis isolates and generated plasmids carrying a pncA mutation.

| Isolate | Mutation | Genotype |
| --- | --- | --- |
| H37Rv | wild type | wild type |
| 3736/04 | A(-11)G | A→G |
| 10299/02 | T(-7)C | T→C |
| 2825/00 | Ile6Thr | A(T/C)C |
| 9008/03 | Asp12Glu | GA(C/G) |
| pAla25Ala | Ala25Ala | GC(C/A) |
| 2483/03 | Leu27Pro | C(T/C)G |
| 6103/09 | Ile31Ser | A(T/G)C |
| 3151/08 | Ala46Ala | GC(A/G) |
| 4724/03 | Thr47Ala | (A/G)CC |
| 9044/05 | Lys48Thr | A(A/C)G |
| 8092/01 | His51Arg | C(A/G)C |
| 4258/00 | His57Asp | (C/G)AC |
| 7683/04 | Ser59Pro | (T/C)CC |
| 4968/03 | Pro62Leu | C(C/T)G |
| 8869/01 | Asp63Ala | G(A/C)C |
| 4897/05 | Tyr64Asp | (T/G)AT |
| 9976/05 | Ser65Pro | (T/C)CC |
| 1879/10 | Ser67Pro | (T/C)CG |
| 8671/04 | Trp68Arg | (T/C)GG |
| 5686/09 | His71Arg | C(A/G)T |
| pSer74Ser | Ser74Ser | AG(C/T) |
| 5158/12 | Thr76Pro | (A/C)CT |
| pLeu116Arg | Leu116Arg | C(T/G)G |
| 6691/04 | Trp119STOP | TG(G/A) |
| 5246/09 | Gln122STOP | (C/T)AA |
| 11234/09 | Del Pos 125-130 | GTC GAT GAG GTC GAT GTG → GT- --- --- --- --- --G |
| 9975/05 | Val128Phe | (G/T)TC |
| 121/04 | Del Pos 129 & 130 | GAT GTG → G-- --G |
| 1202/10 | Gly132Ser | (G/A)GT |
| 10735/04 | Ile133Thr | A(T/C)T |
| 853/07 | Asp136His | (G/C)AT |
| 8131/04 | His137Cys | C(A/G)T |
| 10532/05 | Gln141Pro | C(A/C)G |
| pAsn147Asn | Asn147Asn | G(T/C)A |
| 5073/09 | Arg154Gly | (A/G)GG |
| 3671/04 | Val155Gly | G(T/G)G |
| 2822/06 | Leu159Arg | C(T/G)G |
| 12657/03 | Gly162Asp | G(G/A)T |
| 8655/04 | Val163Ala | G(T/C)G |
| 6704/99 | Ser164Pro | (T/C)CG |

Evaluation of clinical M. tuberculosis isolates from Swaziland.

| # | Isolate | Mutation | DST | Results of the melting curve assay | | |
| --- | --- | --- | --- | --- | --- | --- |
|  |  |  |  | Wild type | Mutant | Mutation detection in the following range |
| 1 | 4248-09 | H51D | r |  | x | c50 to c52 (2.b) |
| 2 | 4249-09 | WT | NA | x |  | --- |
| 3 | 4250-09 | WT | NA | x |  | --- |
| 4 | 4252-09 | G132S | r |  | x | c130 to c135 (2.b) |
| 5 | 4253-09 | WT | NA | x |  | --- |
| 6 | 4255-09 | WT | NA | x |  | --- |
| 7 | 4256-09 | WT | NA | x |  | --- |
| 8 | 4257-09 | WT | NA | x |  | --- |
| 9 | 4258-09 | WT | NA | x |  | --- |
| 10 | 4259-09 | WT | NA | x |  | --- |
| 11 | 4261-09 | WT | NA | x |  | --- |
| 12 | 4262-09 | WT | NA | x |  | --- |
| 13 | 4267-09 | WT | NA | x |  | --- |
| 14 | 4268-09 | WT | NA | x |  | --- |
| 15 | 4269-09 | WT | NA | x |  | --- |
| 16 | 4270-09 | WT | NA | x |  | --- |
| 17 | 4271-09 | WT | NA | x |  | --- |
| 18 | 4272-09 | WT | NA | x |  | --- |
| 19 | 4273-09 | WT | NA | x |  | --- |
| 20 | 4275-09 | WT | NA | x |  | --- |
| 21 | 4276-09 | WT | NA | x |  | --- |
| 22 | 4277-09 | WT | NA | x |  | --- |
| 23 | 4283-09 | T135P | r |  | x | c130 to c137 (1.b) |
| 24 | 4285-09 | WT | NA | x |  | --- |
| 25 | 4286-09 | WT | NA | x |  | --- |
| 26 | 4287-09 | WT | NA | x |  | --- |
| 27 | 4288-09 | L19P | s |  | x | c17 (2.b) to c21 |
| 28 | 4289-09 | M175T | r | x |  | --- |
| 29 | 4290-09 | WT | NA | x |  | --- |
| 30 | 4291-09 | WT | NA | x |  | --- |
| 31 | 4293-09 | WT | NA | x |  | --- |
| 32 | 4294-09 | WT | NA | x |  | --- |
| 33 | 4295-09 | WT | NA | x |  | --- |
| 34 | 4296-09 | WT | NA | x |  | --- |
| 35 | 4297-09 | WT | NA | x |  | --- |
| 36 | 4298-09 | H51D | r |  | x | c50 to c52 (2.b) |
| 37 | 4300-09 | WT | NA | x |  | --- |
| 38 | 4301-09 | WT | NA | x |  | --- |
| 39 | 4328-09 | WT | NA | x |  | --- |
| 40 | 4330-09 | G97D | r | x |  | --- |
| 41 | 4333-09 | WT | NA | x |  | --- |
| 42 | 4335-09 | WT | NA | x |  | --- |
| 43 | 4337-09 | WT | NA | x |  | --- |
| 44 | 4339-09 | G97D | r | x |  | --- |
| 45 | 4343-09 | WT | NA | x |  | --- |
| 46 | 4344-09 | WT | NA | x |  | --- |
| 47 | 4347-09 | WT | NA | x |  | --- |
| 48 | 4687-09 | WT | s | x |  | --- |
| 49 | 4688-09 | WT | NA | x |  | --- |
| 50 | 4691-09 | WT | NA | x |  | --- |
| 51 | 4695-09 | WT | NA | x |  | --- |
| 52 | 4696-09 | WT | NA | x |  | --- |
| 53 | 4697-09 | WT | NA | x |  | --- |
| 54 | 4699-09 | WT | NA | x |  | --- |
| 55 | 4700-09 | H51D | r |  | x | c50 to c52 (2.b) |
| 56 | 4703-09 | WT | NA | x |  | --- |
| 57 | 4709-09 | H51D | r |  | x | c50 to c52 (2.b) |
| 58 | 4711-09 | WT | NA | x |  | --- |
| 59 | 4712-09 | G132S | r |  | x | c130 to c135 (2.b) |
| 60 | 4713-09 | R154G | borderline |  | x | c152 to c155 |
| 61 | 4716-09 | WT | NA | x |  | --- |
| 62 | 4721-09 | H51D | r |  | x | c50 to c52 (2.b) |
| 63 | 4722-09 | WT | NA | x |  | --- |
| 64 | 4723-09 | H51D | r |  | x | c50 to c52 (2.b) |
| 65 | 4724-09 | H51D | r |  | x | c50 to c52 (2.b) |
| 66 | 4725-09 | H51D | r |  | x | c50 to c52 (2.b) |
| 67 | 4727-09 | WT | NA | x |  | --- |
| 68 | 4730-09 | WT | NA | x |  | --- |
| 69 | 4731-09 | WT | NA | x |  | --- |
| 70 | 4735-09 | WT | NA | x |  | --- |
| 71 | 5069-09 | R154G | r |  | x | c152 to c156 |
| 72 | 5072-09 | WT | NA | x |  | --- |
| 73 | 5073-09 | R154G | borderline |  | x | c152 to c156 |
| 74 | 5075-09 | WT | NA | x |  | --- |
| 75 | 5076-09 | WT | NA | x |  | --- |
| 76 | 5078-09 | WT | NA | x |  | --- |
| 77 | 5079-09 | WT | NA | x |  | --- |
| 78 | 5080-09 | Q122STOP | r |  | x | c120 (3.b) to c123 |
| 79 | 5083-09 | WT | NA | x |  | --- |
| 80 | 5088-09 | H51D | r |  | x | c50 to c52 (2.b) |
| 81 | 5093-09 | WT | NA | x |  | --- |
| 82 | 5099-09 | WT | NA | x |  | --- |
| 83 | 5100-09 | L151S | r |  | x | c149 (3.b) to c152 (2.b) |
| 84 | 5102-09 | WT | NA | x |  | --- |
| 85 | 5104-09 | WT | s | x |  | --- |
| 86 | 5105-09 | WT | NA | x |  | --- |
| 87 | 5106-09 | Del125-129 | r |  | x | c122 to c131 |
| 88 | 5107-09 | WT | NA | x |  | --- |
| 89 | 5108-09 | WT | NA | x |  | --- |
| 90 | 5109-09 | H51D | r |  | x | c50 to c52 (2.b) |
| 91 | 5111-09 | Delhi/CAS | NA |  | x | c64 to c68 (2.b) |
| 92 | 5114-09 | D136A | r |  | x | c132 (2.b) to c139 |
| 93 | 5115-09 | WT | NA | x |  | --- |
| 94 | 5117-09 | L35R | NA | x |  | --- |
| 95 | 5118-09 | WT | NA | x |  | --- |
| 96 | 5121-09 | WT | NA | x |  | --- |
| 97 | 5122-09 | WT | NA | x |  | --- |
| 98 | 5126-09 | WT | NA | x |  | --- |
| 99 | 5130-09 | WT | NA | x |  | --- |
| 100 | 5146-09 | S59P | r |  | x | c57 to c61 (2.b) |
| 101 | 5148-09 | WT | NA | x |  | --- |
| 102 | 5149-09 | T114M | borderline | x |  | ---- |
| 103 | 5239-09 | WT | NA | x |  | --- |
| 104 | 5241-09 | WT | s | x |  | --- |
| 105 | 5243-09 | WT | NA | x |  | --- |
| 106 | 5246-09 | Q122STOP | r |  | x | c120 (3.b) to c123 |
| 107 | 5250-09 | WT | NA | x |  | --- |
| 108 | 5251-09 | WT | s | x |  | --- |
| 109 | 5256-09 | L35R | NA | x |  | --- |
| 110 | 5264-09 | WT | NA | x |  | --- |
| 111 | 5268-09 | WT | NA | x |  | --- |
| 112 | 5269-09 | WT | NA | x |  | --- |
| 113 | 5273-09 | WT | NA | x |  | --- |
| 114 | 5274-09 | WT | s | x |  | --- |
| 115 | 5275-09 | H51D | r |  | x | c50 to c52 (2.b) |
| 116 | 5276-09 | WT | NA | x |  | --- |
| 117 | 5282-09 | WT | NA | x |  | --- |
| 118 | 5287-09 | WT | NA | x |  | --- |
| 119 | 5537-09 | WT | s | x |  | --- |
| 120 | 5539-09 | WT | NA | x |  | --- |
| 121 | 5544-09 | WT | NA | x |  | --- |
| 122 | 5549-09 | WT | NA | x |  | --- |
| 123 | 5551-09 | WT | NA | x |  | --- |
| 124 | 5554-09 | WT | NA | x |  | --- |
| 125 | 5556-09 | WT | NA | x |  | --- |
| 126 | 5558-09 | Q10R | r |  | x | c7 to c15 (2.b) |
| 127 | 5569-09 | WT | NA | x |  | --- |
| 128 | 5572-09 | WT | NA | x |  | --- |
| 129 | 5579-09 | H51D | r |  | x | c50 to c52 (2.b) |
| 130 | 5582-09 | WT | NA | x |  | --- |
| 131 | 5737-09 | WT | NA | x |  | --- |
| 132 | 5741-09 | WT | s | x |  | --- |
| 133 | 5743-09 | WT | NA | x |  | --- |
| 134 | 5746-09 | WT | NA | x |  | --- |
| 135 | 5748-09 | G97D | r | x |  | --- |
| 136 | 5750-09 | WT | r | x |  | --- |
| 137 | 5752-09 | WT | NA | x |  | --- |
| 138 | 5753-09 | H51D | r |  | x | c50 to c52 (2.b) |
| 139 | 5755-09 | T135P | r |  | x | c130 to c137 (1.b) |
| 140 | 5758-09 | WT | s | x |  | --- |
| 141 | 5759-09 | WT | NA | x |  | --- |
| 142 | 5771-09 | WT | NA | x |  | --- |
| 143 | 5772-09 | R154G | r |  | x | c152 to c156 |
| 144 | 5797-09 | WT | s | x |  | --- |
| 145 | 6059-09 | R154G | r |  | x | c152 to c156 |
| 146 | 6070-09 | WT | NA | x |  | --- |
| 147 | 6085-09 | WT | NA | x |  | --- |
| 148 | 6087-09 | WT | NA | x |  | --- |
| 149 | 6243-09 | WT | NA | x |  | --- |
| 150 | 6247-09 | WT | s | x |  | --- |
| 151 | 6249-09 | WT | NA | x |  | --- |
| 152 | 6251-09 | WT | NA | x |  | --- |
| 153 | 6255-09 | R154G | r |  | x | c152 to c156 |
| 154 | 6256-09 | WT | NA | x |  | --- |
| 155 | 6258-09 | WT | NA | x |  | --- |
| 156 | 6263-09 | WT | NA | x |  | --- |
| 157 | 6265-09 | WT | NA | x |  | --- |
| 158 | 6266-09 | WT | s | x |  | --- |
| 159 | 6267-09 | R154G | r |  | x | c152 to c156 |
| 160 | 6268-09 | WT | NA | x |  | --- |
| 161 | 6269-09 | H71Y | r |  | x | c68 (3.b) to c73 |
| 162 | 6637-09 | Q10R | r |  | x | c4 (2.b) to c15 (2.b) |
| 163 | 6643-09 | WT | NA | x |  | --- |
| 164 | 6652-09 | R154G | r |  | x | c152 to c156 |
| 165 | 6653-09 | WT | NA | x |  | --- |
| 166 | 6944-09 | H51D | r |  | x | c50 to c52 (2.b) |
| 167 | 6945-09 | G97D | r | x |  | --- |
| 168 | 6948-09 | WT | NA | x |  | --- |
| 169 | 6949-09 | H51D | r |  | x | c50 to c52 (2.b) |
| 170 | 6955-09 | H51D | r |  | x | c50 to c52 (2.b) |
| 171 | 7171-09 | R154G | borderline |  | x | c152 to c156 |
| 172 | 7172-09 | WT | NA | x |  | --- |
| 173 | 7173-09 | R154G | borderline |  | x | c152 to c156 |
| 174 | 7175-09 | H51D | r |  | x | c50 to c52 (2.b) |
| 175 | 7178-09 | R154G | borderline |  | x | c152 to c156 |
| 176 | 7179-09 | G97D | r | x |  | --- |
| 177 | 7185-09 | H51D | r |  | x | c50 to c52 (2.b) |
| 178 | 7186-09 | G97C | r | x |  | --- |
| 179 | 7187-09 | WT | NA | x |  | --- |
| 180 | 7192-09 | WT | s | x |  | --- |
| 181 | 7699-09 | Delhi/CAS | NA |  | x | c64 to c68 (2.b) |
| 182 | 7700-09 | H51D | r |  | x | c50 to c52 (2.b) |
| 183 | 7703-09 | H51D | r |  | x | c50 to c52 (2.b) |
| 184 | 7704-09 | R154G | r |  | x | c152 to c156 |
| 185 | 7708-09 | L151S | borderline |  | x | c149 (3.b) to c152 (2.b) |
| 186 | 7712-09 | WT | s | x |  | --- |
| 187 | 7713-09 | WT | s | x |  | --- |
| 188 | 7716-09 | R154G | borderline |  | x | c152 to c156 |
| 189 | 8302-09 | R154G | borderline |  | x | c152 to c156 |
| 190 | 8304-09 | WT | s | x |  | --- |
| 191 | 8313-09 | WT | s | x |  | --- |
| 192 | 8316-09 | WT | NA | x |  | --- |
| 193 | 8576-09 | Q122STOP | r |  | x | c120 (3.b) to c123 |
| 194 | 8580-09 | WT | NA | x |  | --- |
| 195 | 8581-09 | L151S | r |  | x | c149 (3.b) to c152 (2.b) |
| 196 | 8585-09 | K96R | r | x |  | --- |
| 197 | 8587-09 | WT | NA | x |  | --- |
| 198 | 8591-09 | R154G | borderline |  | x | c152 to c156 |
| 199 | 8592-09 | WT | NA | x |  | --- |
| 200 | 8779-09 | R154G | borderline |  | x | c152 to c156 |
| 201 | 8781-09 | H51D | r |  | x | c50 to c52 (2.b) |
| 202 | 8783-09 | Del125-129 | r |  | x | c122 to c131 |
| 203 | 8784-09 | L151S | r |  | x | c149 (3.b) to c152 (2.b) |
| 204 | 8786-09 | WT | s | x |  | --- |
| 205 | 9080-09 | H51D | r |  | x | c50 to c52 (2.b) |
| 206 | 9081-09 | H51D | r |  | x | c50 to c52 (2.b) |
| 207 | 9085-09 | WT | s | x |  | --- |
| 208 | 9562-09 | R154G | r |  | x | c152 to c156 |
| 209 | 9564-09 | G97C | r | x |  | --- |
| 210 | 9565-09 | WT | NA | x |  | --- |
| 211 | 10108-09 | H51D | r |  | x | c50 to c52 (2.b) |
| 212 | 10114-09 | G97C | r | x |  | --- |
| 213 | 10115-09 | H51D | r |  | x | c50 to c52 (2.b) |
| 214 | 10116-09 | WT | NA | x |  | --- |
| 215 | 10121-09 | R154G | r |  | x | c152 to c156 |
| 216 | 10292-09 | WT | NA | x |  | --- |
| 217 | 10294-09 | H51D | r |  | x | c50 to c52 (2.b) |
| 218 | 10298-09 | H51D | r |  | x | c50 to c52 (2.b) |
| 219 | 10301-09 | WT | s | x |  | --- |
| 220 | 10436-09 | H51D | r |  | x | c50 to c52 (2.b) |
| 221 | 10438-09 | H51D | r |  | x | c50 to c52 (2.b) |
| 222 | 10442-09 | G97C | r | x |  | --- |
| 223 | 10451-09 | H51D | r |  | x | c50 to c52 (2.b) |
| 224 | 10452-09 | H51D | r |  | x | c50 to c52 (2.b) |
| 225 | 10453-09 | WT | s | x |  | --- |
| 226 | 10504-09 | G97C | r | x |  | --- |
| 227 | 10505-09 | R154G | borderline |  | x | c152 to c156 |
| 228 | 10507-09 | -11 A→C | r |  | x | nt -14 to nt -6 |
| 229 | 10512-09 | G97D | r | x |  | --- |
| 230 | 10513-09 | WT | NA | x |  | --- |
| 231 | 10701-09 | H51D | r |  | x | c50 to c52 (2.b) |
| 232 | 10702-09 | R154G | borderline |  | x | c152 to c156 |
| 233 | 10703-09 | L151S | s |  | x | c149 (3.b) to c152 (2.b) |
| 234 | 10704-09 | H51D | r |  | x | c50 to c52 (2.b) |
| 235 | 10706-09 | R154G | borderline |  | x | c152 to c156 |
| 236 | 11015-09 | H51D | r |  | x | c50 to c52 (2.b) |
| 237 | 11016-09 | R154G | borderline |  | x | c152 to c156 |
| 238 | 11020-09 | G97C | r | x |  | --- |
| 239 | 11023-09 | H51D | r |  | x | c50 to c52 (2.b) |
| 240 | 11234-09 | Del125-129 | r |  | x | c122 to c131 |
| 241 | 11236-09 | G97D | r | x |  | --- |
| 242 | 11237-09 | Del125-129 | r |  | x | c122 to c131 |
| 243 | 11238-09 | Del125-129 | r |  | x | c122 to c131 |
| 244 | 111-10 | L151S | r |  | x | c149 (3.b) to c152 (2.b) |
| 245 | 113-10 | T135P | r |  | x | c130 to c137 (1.b) |
| 246 | 117-10 | H71Y | r |  | x | c68 (3.b) to c73 |
| 247 | 118-10 | WT | s | x |  | --- |
| 248 | 119-10 | R154G | r |  | x | c152 to c156 |
| 249 | 173-10 | WT | NA | x |  | --- |
| 250 | 175-10 | Del125-129 | r |  | x | c122 to c131 |
| 251 | 177-10 | WT | NA | x |  | --- |
| 252 | 431-10 | H51D | r |  | x | c50 to c52 (2.b) |
| 253 | 433-10 | H51D | r |  | x | c50 to c52 (2.b) |
| 254 | 596-10 | R154G | borderline |  | x | c152 to c156 |
| 255 | 597-10 | H51D | r |  | x | c50 to c52 (2.b) |
| 256 | 601-10 | R154G | r |  | x | c152 to c156 |
| 257 | 603-10 | Q122STOP | r |  | x | c120 (3.b) to c123 |
| 258 | 1053-10 | R154G | borderline |  | x | c152 to c156 |
| 259 | 1057-10 | WT | s | x |  | --- |
| 260 | 11239-09 | G97D | r | x |  | --- |
| 261 | 1060-10 | WT | NA | x |  | --- |
| 262 | 1207-10 | WT | NA | x |  | --- |
| 263 | 1215-10 | WT | NA | x |  | --- |
| 264 | 1210-10 | H51D | r |  | x | c50 to c52 (2.b) |
| 265 | 1213-10 | WT | s | x |  | --- |
| 266 | 110-10 | WT | NA | x |  | --- |
| 267 | 1054-10 | WT | s | x |  | --- |
| 268 | 1202-10 | G132S | r |  | x | c130 to c135 (2.b) |
| 269 | 438-10 | H51D | r |  | x | c50 to c52 (2.b) |
| 270 | 6078-09 | Y103STOP | r | x |  | --- |
| 271 | 604-10 | WT | s | x |  | --- |

b – base, c – codon, s – susceptible, r – resistant, borderline - detectable growth below the resistant threshold, NA – not applied for fully first-line susceptible isolates, WT – wild type
