# Supplementary material for: Rapid microarray-based assay for detection of pyrazinamide resistant Mycobacterium tuberculosis
Source: Diagn Microbiol Infect Dis. 2019 Jun;94(2):147–54. doi: 10.1016/j.diagmicrobio.2018.12.011 (PMC6531379; doi:10.1016/j.diagmicrobio.2018.12.011)
Supplement: Table S2 — Primers, probes and TaqMan® probes. [file mmc2.docx]

Table S2. Primers, probes and TaqMan® probes.

*Primers*

| Target | Primer | Sequence (5´-3´) | Product size (bp) | Sequence length (bp) | T_m_ (°C) |
| --- | --- | --- | --- | --- | --- |
| *pncA* A1^a^ | pncA fw1 | GCGTCGGTAGGCAAACTGC | 161 | 19 | 59.4 |
|  | pncA rv1 | TTCGGCCAGGTAGTCGCT |  | 18 | 58.0 |
| *pncA* A2 ^a^ | pncA fw2 | GCGGACTACCATCACGTCGT | 217 | 20 | 59.6 |
|  | pncA rv2 | CGACTCCTTCGAAGCCGCT |  | 19 | 59.7 |
| *pncA* A3 ^a^ | pncA fw3 | CGACGAGAACGGCACGC | 265 | 17 | 59.1 |
|  | pncA rv3 | CCAACAGTTCATCCCGGTTCG |  | 21 | 58.9 |
| process control^b^ | Rand250_fw | TGGTAACCTGGATTCCTAACTGGA | 85 | 24 | 57.9 |
|  | Rand250_rv | ACCGTGTCTGGAATCTCTCTACA |  | 23 | 57.8 |

*^a^* Rv2043c; GenBank accession no. NC_000962.3

*^b^* artificial sequence

*Probes*

| Names | Sequence (5´-3´) | Evalua-tion group | Covered gene region |
| --- | --- | --- | --- |
| hp:pncA_080 | CACCATACGTTCGGGCGACTGCC | 3 | nt -28 to -6 |
| hp:pncA_082 | GCATACGTCCACCATACGTTCGGGCG | 4 | nt -22 to c2 (1.b) |
| hp:pncA_002 | CCCGCATACGTCCACCATACG | 1 | nt -18 to c3 (1.b) |
| hp:pncA_085 | GATCAACGCCCGCATACGTCCACCA | 6 | nt -10 to c5 |
| hp:pncA_004 | GACGATGATCAACGCCCGCAT | 3 | c1 to c7 |
| hp:pncA_088 | CGTCGACGATGATCAACGCCCGCATAC | 6 | nt -2 to c9 (1.b) |
| hp:pncA_090 | TTCTGCACGTCGACGATGATCAACGCC | 5 | c2 (3.b) to c11 (2.b) |
| hp:pncA_092 | AGAAGTCGTTCTGCACGTCGACGATGATCA | 6 | c4 (2.b) to c14 (1.b) |
| hp:pncA_093 | TCGCAGAAGTCGTTCTGCACGTCGAC | 4 | c7 to 15 (2.b) |
| hp:pncA_096 | AGCCACCCTCGCAGAAGTCGTTCTG | 4 | c10 to c18 (1.b) |
| hp:pncA_009 | AGCGAGCCACCCTCGCAGAAG | 5 | c12 (3.b) to c19 (2.b) |
| hp:pncA_010 | TACCGCCAGCGAGCCACCCTC | 6 | c15 to c21 |
| hp:pncA_011 | CACCGGTTACCGCCAGCGAGC | 3 | c17 (2.b) to c24 (1.b) |
| hp:pncA_103 | GGCGCCACCGGTTACCGCC | 3 | c19 (3.b) to c25 |
| hp:pncA_013 | GGCCAGCGCGGCGCCACCGGT | 5 | c22 to c28 |
| hp:pncA_107 | GCGGGCCAGCGCGGC | 2 | c24 (3.b) to c30(1.b) |
| hp:pncA_071 | GCTGATGGCGCGGGCCAGCGC | 6 | c26 to c32 |
| hp:pncA_113^a^ | GGAAGTCCTTGGTCGCCACGACGT | --- | c43 (2.b) to c51 (1.b) |
| hp:pncA_067 | ATGTGGAAGTCCTTGGTTGCC | 2 | c45 (3.b) to c52 (2.b) |
| hp:pncA_015 | GGGTCGATGTGGAAGTCCTTG | 1 | c47 (3.b) to c54 (2.b) |
| hp:pncA_016 | GTCACCCGGGTCGATGTGGAA | 2 | c50 to c56 |
| hp:pncA_017 | AGAAGTGGTCACCCGGGTCGA | 2 | c52 (2.b) to c59 (1.b) |
| hp:pncA_074_His57Asp^b^ | CCGGAGAAGTCGTCACCCGGG | --- | c53 (3.b) to c60 (2.b) |
| hp:pncA_018 | GTGCCGGAGAAGTGGTCACCC | 2 | c54 (3.b) to c61 (2.b) |
| hp:pncA_019 | GTCCGGTGTGCCGGAGAAGTG | 2 | c57 to c63 |
| hp:pncA_020 | AGGAATAGTCCGGTGTGCCGG | 1 | c59 (2.b) to c66 (1.b) |
| hp:pncA_021 | CACGACGAGGAATAGTCCGGT | 1 | c61 (3.b) to c68 (2.b) |
| hp:pncA_137^c^ | GGCCACGACGAAGAATAGTCCGGTGT | --- | c61 to c69 (2.b) |
| hp:pncA_022 | CGGTGGCCACGACGAGGAATA | 2 | c64 to c70 |
| hp:pncA_142 | GACGCAATGCGGTGGCCACGAC | 3 | c66 (3.b) to c73 |
| hp:pncA_024 | CCGCTGACGCAATGCGGTGGC | 2 | c68 (3.b) to c75 (2.b) |
| hp:pncA_025 | GGGAGTACCGCTGACGCAATG | 1 | c71 to c77 |
| hp:pncA_077_Ser74AGC | CGGGAGTACCGCTGACGCAAT | 1 | c71 (2.b) to c78 (1.b) |
| hp:pncA_078_Ser74AGT^d^ | CGGGAGTACCACTGACGCAAT | --- | c71 (2.b) to c78 (1.b) |
| hp:pncA_176 | CGTTGCCGCAGCCAATTCAGCAGTG | 4 | c115 (2.b) to c123 (2.b) |
| hp:pncA_037 | CCGCGTTGCCGCAGCCAATTC | 5 | c117 (3.b) to c124 (2.b) |
| hp:pncA_179 | CGACGCCGCGTTGCCGC | 2 | c120 (3.b) to c126 (1.b) |
| hp:pncA_039 | CGACCTCATCGACGCCGCGTT | 3 | c122 (2.b) to c129 (1.b) |
| hp:pncA_040 | ACCACATCGACCTCATCGACG | 1 | c124 (3.b) to 131 (2.b) |
| hp:pncA_186 | GTGGCAATACCGACCACATCGACCTCATC | 5 | c126 to c135 (2.b) |
| hp:pncA_187 | GATCGGTGGCAATACCGACCACATCGAC | 6 | c128 to c137 (1.b) |
| hp:pncA_190 | CACACAATGATCGGTGGCAATACCGACCAC | 6 | c130 to c139 |
| hp:pncA_191 | GGCGCACACAATGATCGGTGGCAATAC | 5 | c132 (2.b) to c141 (1.b) |
| hp:pncA_194 | GGCCGTCTGGCGCACACAATGATC | 4 | c136 to c143 |
| hp:pncA_046 | TCCTCGGCCGTCTGGCGCACA | 3 | c138 (3.b) to c145 (2.b) |
| hp:pncA_198 | GTACCGCGTCCTCGGCCGTCT | 3 | c141 (2.b) to 148 (1.b) |
| hp:pncA_199 | CATTGCGTACCGCGTCCTCGGC | 3 | c143 to c150 (1.b) |
| hp:pncA_049 | GCCAAGCCATTGCGTACCGCG | 2 | c145 (3.b) to c152 (2.b) |
| hp:pncA_253 | ACCCTGGTGGCCAAGCCATTGCGT | 3 | c149 to c155 (2.b) |
| hp:pncA_206 | CAGCACCCTGGTGGCCAAGCCA | 5 | c149 (3.b) to c156 |
| hp:pncA_255 | TCAGGTCCACCAGCACCCTGGTGGC | 4 | c152 to c160 (1.b) |
| hp:pncA_053 | CGCTGTCAGGTCCACCAGCAC | 2 | c155 to c161 |
| hp:pncA_054 | ACACACCCGCTGTCAGGTCCA | 2 | c157 (2.b) to c164 (1.b) |
| hp:pncA_244 | ATCGGCCGACACACCCGCTGTCA | 5 | c159 (2.b) to c166 |
| hp:pncA_258 | CGGTGGTATCGGCCGACACACCCGC | 5 | c161 to c169 (1.b) |

nt – nucleotide; c – codon; b – base pair

^a^ probe for the detection of *Mycobacterium canettii*

^b^ probe for the detection of *Mycobacterium bovis*

^c^ probe for the detection of *Mycobacterium Delhi/CAS* lineage

^d^ probe for the detection of a silent mutation at position 74

*TaqMan® probes*

| TaqMan® probe | Sequence (5´-3´) | 5´ Modification | 3´ Modification |
| --- | --- | --- | --- |
| *pncA* A1 | CGACGTGCAGAACGACTTCTGCGA | FAM | BHQ-1 |
| *pncA* A2 | CCTCGTCGTGGCCACCGCA | Cy5 | BHQ-3 |
| *pncA* A3 | AGGACGCGGTACGCAATGGCT | Texas Red | BHQ-2 |
| Rand 250 | ACTGCGAACATTACAACTTGGAGCGT | JOE | BHQ-1 |
