# Supplementary material for: Rapid microarray-based assay for detection of pyrazinamide resistant Mycobacterium tuberculosis
Source: Diagn Microbiol Infect Dis. 2019 Jun;94(2):147–54. doi: 10.1016/j.diagmicrobio.2018.12.011 (PMC6531379; doi:10.1016/j.diagmicrobio.2018.12.011)
Supplement: Table S3 — Test results for the analysis of different pncA isolates using the melting curve assay. [file mmc3.docx]

Table S3. Test results for the analysis of different pncA isolates using the melting curve assay.

| **Isolate** | **Affected**  **Amplicon** | **Mutation** | **Hybridization probes (hp) *pncA* A1** | | | | | | | | | | | | | | | | |
| --- | --- | --- | --- | --- | --- | --- | --- | --- | --- | --- | --- | --- | --- | --- | --- | --- | --- | --- | --- |
|  |  |  | **hp:pncA_080_rc** | **hp:pncA_082_rc** | **hp:pncA_002_rc** | **hp:pncA_085_rc** | **hp:pncA_004_rc** | **hp:pncA_088_rc** | **hp:pncA_090_rc** | **hp:pncA_092_rc** | **hp:pncA_093_rc** | **hp:pncA_096_rc** | **hp:pncA_009_rc** | **hp:pncA_010_rc** | **hp:pncA_011_rc** | **hp:pncA_103_rc** | **hp:pncA_013_rc** | **hp:pncA_107_rc** | **hp:pncA_071_rc** |
| 3736/04 | 1 | A(-11)G | x | x | x |  |  |  |  |  |  |  |  |  |  |  |  |  |  |
| 10299/02 | 1 | T(-7)C | x | x | x | x |  |  |  |  |  |  |  |  |  |  |  |  |  |
| 2825/00 | 1 | Ile6Thr |  |  |  |  | x | x | x | x |  |  |  |  |  |  |  |  |  |
| 9008/03 | 1 | Asp12Glu |  |  |  |  |  |  |  | x | x | x | x |  |  |  |  |  |  |
| (artificial) | 1 | pAla25Ala |  |  |  |  |  |  |  |  |  |  |  |  |  | x | x | x |  |
| 2483/03 | 1 | Leu27Pro |  |  |  |  |  |  |  |  |  |  |  |  |  |  | x | x | x |
| 6103/09 | 1 | Ile31Ser |  |  |  |  |  |  |  |  |  |  |  |  |  |  |  |  | x |
| 3151/08 | 2 | Ala46Ala |  |  |  |  |  |  |  |  |  |  |  |  |  |  |  |  |  |
| 4724/03 | 2 | Thr47Ala |  |  |  |  |  |  |  |  |  |  |  |  |  |  |  |  |  |
| 9044/05 | 2 | Lys48Thr |  |  |  |  |  |  |  |  |  |  |  |  |  |  |  |  |  |
| 8092/01 | 2 | His51Arg |  |  |  |  |  |  |  |  |  |  |  |  |  |  |  |  |  |
| 4258/00 | 2 | His57Asp |  |  |  |  |  |  |  |  |  |  |  |  |  |  |  |  |  |
| 7683/04 | 2 | Ser59Pro |  |  |  |  |  |  |  |  |  |  |  |  |  |  |  |  |  |
| 4968/03 | 2 | Pro62Leu |  |  |  |  |  |  |  |  |  |  |  |  |  |  |  |  |  |
| 8869/01 | 2 | Asp63Ala |  |  |  |  |  |  |  |  |  |  |  |  |  |  |  |  |  |
| 4897/05 | 2 | Tyr64Asp |  |  |  |  |  |  |  |  |  |  |  |  |  |  |  |  |  |
| 9976/05 | 2 | Ser65Pro |  |  |  |  |  |  |  |  |  |  |  |  |  |  |  |  |  |
| 1879/10 | 2 | Ser67Pro |  |  |  |  |  |  |  |  |  |  |  |  |  |  |  |  |  |
| 8671/04 | 2 | Trp68Arg |  |  |  |  |  |  |  |  |  |  |  |  |  |  |  |  |  |
| 5686/09 | 2 | His71Arg |  |  |  |  |  |  |  |  |  |  |  |  |  |  |  |  |  |
| (artificial) | 2 | pSer74Ser |  |  |  |  |  |  |  |  |  |  |  |  |  |  |  |  |  |
| 5158/12 | 2 | Thr76Pro |  |  |  |  |  |  |  |  |  |  |  |  |  |  |  |  |  |
| (artificial) | 3 | Leu116Arg |  |  |  |  |  |  |  |  |  |  |  |  |  |  |  |  |  |
| 6691/04 | 3 | Trp119STOP |  |  |  |  |  |  |  |  |  |  |  |  |  |  |  |  |  |
| 5246/09 | 3 | Gln122stopp |  |  |  |  |  |  |  |  |  |  |  |  |  |  |  |  |  |
| 11234/09 | 3 | Deletion Pos 125-130 |  |  |  |  |  |  |  |  |  |  |  |  |  |  |  |  |  |
| 9975/05 | 3 | Val128Phe |  |  |  |  |  |  |  |  |  |  |  |  |  |  |  |  |  |
| 121/04 | 3 | Del Pos 129 und 130 |  |  |  |  |  |  |  |  |  |  |  |  |  |  |  |  |  |
| 1202/10 | 3 | Gly132Ser |  |  |  |  |  |  |  |  |  |  |  |  |  |  |  |  |  |
| 10735/04 | 3 | Ile133Thr |  |  |  |  |  |  |  |  |  |  |  |  |  |  |  |  |  |
| 853/07 | 3 | Asp136His |  |  |  |  |  |  |  |  |  |  |  |  |  |  |  |  |  |
| 8131/04 | 3 | His137Cys |  |  |  |  |  |  |  |  |  |  |  |  |  |  |  |  |  |
| 10532/05 | 3 | Gln141Pro |  |  |  |  |  |  |  |  |  |  |  |  |  |  |  |  |  |
| (artificial) | 3 | Asn147Asn |  |  |  |  |  |  |  |  |  |  |  |  |  |  |  |  |  |
| 5073/09 | 3 | Arg154Gly |  |  |  |  |  |  |  |  |  |  |  |  |  |  |  |  |  |
| 3671/04 | 3 | Val155Gly |  |  |  |  |  |  |  |  |  |  |  |  |  |  |  |  |  |
| 2822/06 | 3 | Leu159Arg |  |  |  |  |  |  |  |  |  |  |  |  |  |  |  |  |  |
| 12657/03 | 3 | Gly162Asp |  |  |  |  |  |  |  |  |  |  |  |  |  |  |  |  |  |
| 8655/04 | 3 | Val163Ala |  |  |  |  |  |  |  |  |  |  |  |  |  |  |  |  |  |
| 6704/99 | 3 | Ser164Pro |  |  |  |  |  |  |  |  |  |  |  |  |  |  |  |  |  |

| **Isolate** | **Affected**  **Amplicon** | **Mutation** | **Hybridization probes (hp) *pncA* A2** | | | | | | | | | | | | | | | | |  | | | |
| --- | --- | --- | --- | --- | --- | --- | --- | --- | --- | --- | --- | --- | --- | --- | --- | --- | --- | --- | --- | --- | --- | --- | --- |
|  |  |  | **hp:pncA_113_rc** | **hp:pncA_067_rc** | **hp:pncA_015_as** | **hp:pncA_016_as** | **hp:pncA_017_as** | **hp:pncA_074_His57Asp_rc** | **hp:pncA_018_as** | **hp:pncA_019_as** | **hp:pncA_020_as** | **hp:pncA_021_as** | **hp:pncA_137_rc** | **hp:pncA_022_as** | **hp:pncA_142_rc** | **hp:pncA_024_as** | **hp:pncA_025_as** | **hp:pncA_077_Ser74AGC_rc** | **hp:pncA_078_Ser74AGT_rc** | **SNP M. canettii** | **SNP M. bovis** | **SNP Delhi/CAS** | **SNP silent 74** |
| 3736/04 | 1 | A(-11)G |  |  |  |  |  |  |  |  |  |  |  |  |  |  |  |  |  | wt | wt | wt | wt |
| 10299/02 | 1 | T(-7)C |  |  |  |  |  |  |  |  |  |  |  |  |  |  |  |  |  | wt | wt | wt | wt |
| 2825/00 | 1 | Ile6Thr |  |  |  |  |  |  |  |  |  |  |  |  |  |  |  |  |  | wt | wt | wt | wt |
| 9008/03 | 1 | Asp12Glu |  |  |  |  |  |  |  |  |  |  |  |  |  |  |  |  |  | wt | wt | wt | wt |
| (artificial) | 1 | pAla25Ala |  |  |  |  |  |  |  |  |  |  |  |  |  |  |  |  |  | wt | wt | wt | wt |
| 2483/03 | 1 | Leu27Pro |  |  |  |  |  |  |  |  |  |  |  |  |  |  |  |  |  | wt | wt | wt | wt |
| 6103/09 | 1 | Ile31Ser |  |  |  |  |  |  |  |  |  |  |  |  |  |  |  |  |  | wt | wt | wt | wt |
| 3151/08 | 2 | Ala46Ala |  | x |  |  |  |  |  |  |  |  |  |  |  |  |  |  |  | mut | wt | wt | wt |
| 4724/03 | 2 | Thr47Ala |  | x | x |  |  |  |  |  |  |  |  |  |  |  |  |  |  | mut | wt | wt | wt |
| 9044/05 | 2 | Lys48Thr |  | x | x |  |  |  |  |  |  |  |  |  |  |  |  |  |  | wt | wt | wt | wt |
| 8092/01 | 2 | His51Arg |  | x | x | x |  |  |  |  |  |  |  |  |  |  |  |  |  | wt | wt | wt | wt |
| 4258/00 | 2 | His57Asp |  |  |  |  | x |  | x | x |  |  |  |  |  |  |  |  |  | wt | mut | wt | wt |
| 7683/04 | 2 | Ser59Pro |  |  |  |  | x |  | x | x | x |  |  |  |  |  |  |  |  | wt | wt | wt | wt |
| 4968/03 | 2 | Pro62Leu |  |  |  |  |  |  |  | x | x | x |  |  |  |  |  |  |  | wt | wt | wt | wt |
| 8869/01 | 2 | Asp63Ala |  |  |  |  |  |  |  | x | x | x |  |  |  |  |  |  |  | wt | wt | wt | wt |
| 4897/05 | 2 | Tyr64Asp |  |  |  |  |  |  |  |  | x | x |  | x |  |  |  |  |  | wt | wt | wt | wt |
| 9976/05 | 2 | Ser65Pro |  |  |  |  |  |  |  |  | x | x |  | x |  |  |  |  |  | wt | wt | wt | wt |
| 1879/10 | 2 | Ser67Pro |  |  |  |  |  |  |  |  |  | x |  | x | x |  |  |  |  | wt | wt | wt | wt |
| 8671/04 | 2 | Trp68Arg |  |  |  |  |  |  |  |  |  | x |  | x | x | x |  |  |  | wt | wt | wt | wt |
| 5686/09 | 2 | His71Arg |  |  |  |  |  |  |  |  |  |  |  |  | x | x | x | x |  | wt | wt | wt | wt |
| (artificial) | 2 | pSer74Ser |  |  |  |  |  |  |  |  |  |  |  |  |  | x | x | x |  | wt | wt | wt | mut |
| 5158/12 | 2 | Thr76Pro |  |  |  |  |  |  |  |  |  |  |  |  |  |  | x | x |  | wt | wt | wt | wt |
| (artificial) | 3 | Leu116Arg |  |  |  |  |  |  |  |  |  |  |  |  |  |  |  |  |  | wt | wt | wt | wt |
| 6691/04 | 3 | Trp119STOP |  |  |  |  |  |  |  |  |  |  |  |  |  |  |  |  |  | wt | wt | wt | wt |
| 5246/09 | 3 | Gln122stopp |  |  |  |  |  |  |  |  |  |  |  |  |  |  |  |  |  | wt | wt | wt | wt |
| 11234/09 | 3 | Deletion Pos 125-130 |  |  |  |  |  |  |  |  |  |  |  |  |  |  |  |  |  | wt | wt | wt | wt |
| 9975/05 | 3 | Val128Phe |  |  |  |  |  |  |  |  |  |  |  |  |  |  |  |  |  | wt | wt | wt | wt |
| 121/04 | 3 | Del Pos 129 und 130 |  |  |  |  |  |  |  |  |  |  |  |  |  |  |  |  |  | wt | wt | wt | wt |
| 1202/10 | 3 | Gly132Ser |  |  |  |  |  |  |  |  |  |  |  |  |  |  |  |  |  | wt | wt | wt | wt |
| 10735/04 | 3 | Ile133Thr |  |  |  |  |  |  |  |  |  |  |  |  |  |  |  |  |  | wt | wt | wt | wt |
| 853/07 | 3 | Asp136His |  |  |  |  |  |  |  |  |  |  |  |  |  |  |  |  |  | wt | wt | wt | wt |
| 8131/04 | 3 | His137Cys |  |  |  |  |  |  |  |  |  |  |  |  |  |  |  |  |  | wt | wt | wt | wt |
| 10532/05 | 3 | Gln141Pro |  |  |  |  |  |  |  |  |  |  |  |  |  |  |  |  |  | wt | wt | wt | wt |
| (artificial) | 3 | Asn147Asn |  |  |  |  |  |  |  |  |  |  |  |  |  |  |  |  |  | wt | wt | wt | wt |
| 5073/09 | 3 | Arg154Gly |  |  |  |  |  |  |  |  |  |  |  |  |  |  |  |  |  | wt | wt | wt | wt |
| 3671/04 | 3 | Val155Gly |  |  |  |  |  |  |  |  |  |  |  |  |  |  |  |  |  | wt | wt | wt | wt |
| 2822/06 | 3 | Leu159Arg |  |  |  |  |  |  |  |  |  |  |  |  |  |  |  |  |  | wt | wt | wt | wt |
| 12657/03 | 3 | Gly162Asp |  |  |  |  |  |  |  |  |  |  |  |  |  |  |  |  |  | wt | wt | wt | wt |
| 8655/04 | 3 | Val163Ala |  |  |  |  |  |  |  |  |  |  |  |  |  |  |  |  |  | wt | wt | wt | wt |
| 6704/99 | 3 | Ser164Pro |  |  |  |  |  |  |  |  |  |  |  |  |  |  |  |  |  | wt | wt | wt | wt |

| **Isolate** | **Affected**  **Amplicon** | **Mutation** | **Hybridization probes (hp) *pncA* A3** | | | | | | | | | | | | | | | | | | | | |
| --- | --- | --- | --- | --- | --- | --- | --- | --- | --- | --- | --- | --- | --- | --- | --- | --- | --- | --- | --- | --- | --- | --- | --- |
|  |  |  | **hp:pncA_176_rc** | **hp:pncA_037_rc** | **hp:pncA_179_rc** | **hp:pncA_039_rc** | **hp:pncA_040_rc** | **hp:pncA_186_rc** | **hp:pncA_187_rc** | **hp:pncA_190_rc** | **hp:pncA_191_rc** | **hp:pncA_194_rc** | **hp:pncA_046_rc** | **hp:pncA_198_rc** | **hp:pncA_199_rc** | **hp:pncA_049_rc** | **hp:pncA_253_rc** | **hp:pncA_206_rc** | **hp:pncA_255_rc** | **hp:pncA_053_rc** | **hp:pncA_054_rc** | **hp:pncA_244_rc** | **hp:pncA_258_rc** |
| 3736/04 | 1 | A(-11)G |  |  |  |  |  |  |  |  |  |  |  |  |  |  |  |  |  |  |  |  |  |
| 10299/02 | 1 | T(-7)C |  |  |  |  |  |  |  |  |  |  |  |  |  |  |  |  |  |  |  |  |  |
| 2825/00 | 1 | Ile6Thr |  |  |  |  |  |  |  |  |  |  |  |  |  |  |  |  |  |  |  |  |  |
| 9008/03 | 1 | Asp12Glu |  |  |  |  |  |  |  |  |  |  |  |  |  |  |  |  |  |  |  |  |  |
| (artificial) | 1 | pAla25Ala |  |  |  |  |  |  |  |  |  |  |  |  |  |  |  |  |  |  |  |  |  |
| 2483/03 | 1 | Leu27Pro |  |  |  |  |  |  |  |  |  |  |  |  |  |  |  |  |  |  |  |  |  |
| 6103/09 | 1 | Ile31Ser |  |  |  |  |  |  |  |  |  |  |  |  |  |  |  |  |  |  |  |  |  |
| 3151/08 | 2 | Ala46Ala |  |  |  |  |  |  |  |  |  |  |  |  |  |  |  |  |  |  |  |  |  |
| 4724/03 | 2 | Thr47Ala |  |  |  |  |  |  |  |  |  |  |  |  |  |  |  |  |  |  |  |  |  |
| 9044/05 | 2 | Lys48Thr |  |  |  |  |  |  |  |  |  |  |  |  |  |  |  |  |  |  |  |  |  |
| 8092/01 | 2 | His51Arg |  |  |  |  |  |  |  |  |  |  |  |  |  |  |  |  |  |  |  |  |  |
| 4258/00 | 2 | His57Asp |  |  |  |  |  |  |  |  |  |  |  |  |  |  |  |  |  |  |  |  |  |
| 7683/04 | 2 | Ser59Pro |  |  |  |  |  |  |  |  |  |  |  |  |  |  |  |  |  |  |  |  |  |
| 4968/03 | 2 | Pro62Leu |  |  |  |  |  |  |  |  |  |  |  |  |  |  |  |  |  |  |  |  |  |
| 8869/01 | 2 | Asp63Ala |  |  |  |  |  |  |  |  |  |  |  |  |  |  |  |  |  |  |  |  |  |
| 4897/05 | 2 | Tyr64Asp |  |  |  |  |  |  |  |  |  |  |  |  |  |  |  |  |  |  |  |  |  |
| 9976/05 | 2 | Ser65Pro |  |  |  |  |  |  |  |  |  |  |  |  |  |  |  |  |  |  |  |  |  |
| 1879/10 | 2 | Ser67Pro |  |  |  |  |  |  |  |  |  |  |  |  |  |  |  |  |  |  |  |  |  |
| 8671/04 | 2 | Trp68Arg |  |  |  |  |  |  |  |  |  |  |  |  |  |  |  |  |  |  |  |  |  |
| 5686/09 | 2 | His71Arg |  |  |  |  |  |  |  |  |  |  |  |  |  |  |  |  |  |  |  |  |  |
| (artificial) | 2 | pSer74Ser |  |  |  |  |  |  |  |  |  |  |  |  |  |  |  |  |  |  |  |  |  |
| 5158/12 | 2 | Thr76Pro |  |  |  |  |  |  |  |  |  |  |  |  |  |  |  |  |  |  |  |  |  |
| (artificial) | 3 | Leu116Arg | x |  |  |  |  |  |  |  |  |  |  |  |  |  |  |  |  |  |  |  |  |
| 6691/04 | 3 | Trp119STOP | x | x |  |  |  |  |  |  |  |  |  |  |  |  |  |  |  |  |  |  |  |
| 5246/09 | 3 | Gln122stopp | x | x | x | x |  |  |  |  |  |  |  |  |  |  |  |  |  |  |  |  |  |
| 11234/09 | 3 | Deletion Pos 125-130 |  |  | x | x | x | x | x | x |  |  |  |  |  |  |  |  |  |  |  |  |  |
| 9975/05 | 3 | Val128Phe |  |  |  | x | x | x | x |  |  |  |  |  |  |  |  |  |  |  |  |  |  |
| 121/04 | 3 | Del Pos 129 und 130 |  |  |  | x | x | x | x | x |  |  |  |  |  |  |  |  |  |  |  |  |  |
| 1202/10 | 3 | Gly132Ser |  |  |  |  |  | x | x | x |  |  |  |  |  |  |  |  |  |  |  |  |  |
| 10735/04 | 3 | Ile133Thr |  |  |  |  |  | x | x | x | x |  |  |  |  |  |  |  |  |  |  |  |  |
| 853/07 | 3 | Asp136His |  |  |  |  |  |  | x | x | x | x |  |  |  |  |  |  |  |  |  |  |  |
| 8131/04 | 3 | His137Cys |  |  |  |  |  |  | x | x | x | x |  |  |  |  |  |  |  |  |  |  |  |
| 10532/05 | 3 | Gln141Pro |  |  |  |  |  |  |  |  | x | x | x | x |  |  |  |  |  |  |  |  |  |
| (artificial) | 3 | Asn147Asn |  |  |  |  |  |  |  |  |  |  |  | x | x | x | x |  |  |  |  |  |  |
| 5073/09 | 3 | Arg154Gly |  |  |  |  |  |  |  |  |  |  |  |  |  |  | x | x | x |  |  |  |  |
| 3671/04 | 3 | Val155Gly |  |  |  |  |  |  |  |  |  |  |  |  |  |  | x | x | x | x |  |  |  |
| 2822/06 | 3 | Leu159Arg |  |  |  |  |  |  |  |  |  |  |  |  |  |  |  |  | x | x | x | x |  |
| 12657/03 | 3 | Gly162Asp |  |  |  |  |  |  |  |  |  |  |  |  |  |  |  |  |  |  | x | x | x |
| 8655/04 | 3 | Val163Ala |  |  |  |  |  |  |  |  |  |  |  |  |  |  |  |  |  |  | x | x | x |
| 6704/99 | 3 | Ser164Pro |  |  |  |  |  |  |  |  |  |  |  |  |  |  |  |  |  |  | x | x | x |

|  |  | Detection of the outlier |
| --- | --- | --- |
|  |  | Wrong detection |

The table shows the results of different *pncA* isolates (vertical arrangement) for the three amplicons (*pncA* A1, *pncA* A2 and *pncA* A3). The expected theoretical hybridization result based on the *pncA* genotype is indicated by a cross for each array probe. The reaction pattern of *pncA* isolates with the probes are marked as green colored boxes if mutations were detected automatically as an outlier by the defined algorithm. A purple colored box represent probes which were determined wrongly as outliers. The four mutant probes within the amplicon *pncA* A2 are shown as slanting lines and the respective results are given in the last four columns (wt – wild type or mut – mutation).
