# Supplementary material for: Rapid microarray-based assay for detection of pyrazinamide resistant Mycobacterium tuberculosis
Source: Diagn Microbiol Infect Dis. 2019 Jun;94(2):147–54. doi: 10.1016/j.diagmicrobio.2018.12.011 (PMC6531379; doi:10.1016/j.diagmicrobio.2018.12.011)
Supplement: Table S4 — Results of corresponding pairs of genomic DNA and crude culture material from pncA isolates in the melting curve assay. [file mmc4.docx]

Table S4. Results of corresponding pairs of genomic DNA and crude culture material from pncA isolates in the melting curve assay.

| **Isolate** | **Affected Amplicon** | **Mutation** | **Sample type** | **Hybridization probes (hp) *pncA* A1** | | | | | | | | | | | | | | | | |
| --- | --- | --- | --- | --- | --- | --- | --- | --- | --- | --- | --- | --- | --- | --- | --- | --- | --- | --- | --- | --- |
|  |  |  |  | **hp:pncA_080_rc** | **hp:pncA_082_rc** | **hp:pncA_002_rc** | **hp:pncA_085_rc** | **hp:pncA_004_rc** | **hp:pncA_088_rc** | **hp:pncA_090_rc** | **hp:pncA_092_rc** | **hp:pncA_093_rc** | **hp:pncA_096_rc** | **hp:pncA_009_rc** | **hp:pncA_010_rc** | **hp:pncA_011_rc** | **hp:pncA_103_rc** | **hp:pncA_013_rc** | **hp:pncA_107_rc** | **hp:pncA_071_rc** |
| 4711/09 | --- | --- | genomic DNA |  |  |  |  |  |  |  |  |  |  |  |  |  |  |  |  |  |
|  |  |  | crude culture material |  |  |  |  |  |  |  |  |  |  |  |  |  |  |  |  |  |
| 10298/09 | 2 | His51Asp | genomic DNA |  |  |  |  |  |  |  |  |  |  |  |  |  |  |  |  |  |
|  |  |  | crude culture material |  |  |  |  |  |  |  |  |  |  |  |  |  |  |  |  |  |
| 11237/09 | 3 | Del125-129 | genomic DNA |  |  |  |  |  |  |  |  |  |  |  |  |  |  |  |  |  |
|  |  |  | crude culture material |  |  |  |  |  |  |  |  |  |  |  |  |  |  |  |  |  |
| 5114/09 | 3 | Asp136Ala | genomic DNA |  |  |  |  |  |  |  |  |  |  |  |  |  |  |  |  |  |
|  |  |  | crude culture material |  |  |  |  |  |  |  |  |  |  |  |  |  |  |  |  |  |
| 5100/09 | 3 | Leu151Ser | genomic DNA |  |  |  |  |  |  |  |  |  |  |  |  |  |  |  |  |  |
|  |  |  | crude culture material |  |  |  |  |  |  |  |  |  |  |  |  |  |  |  |  |  |
| 5772/09 | 3 | Arg154Gly | genomic DNA |  |  |  |  |  |  |  |  |  |  |  |  |  |  |  |  |  |
|  |  |  | crude culture material |  |  |  |  |  |  |  |  |  |  |  |  |  |  |  |  |  |

| **Isolate** | **Affected Amplicon** | **Mutation** | **Sample type** | **Hybridization probes (hp) *pncA* A2** | | | | | | | | | | | | | | | | |  | | | |
| --- | --- | --- | --- | --- | --- | --- | --- | --- | --- | --- | --- | --- | --- | --- | --- | --- | --- | --- | --- | --- | --- | --- | --- | --- |
|  |  |  |  | **hp:pncA_113_rc** | **hp:pncA_067_rc** | **hp:pncA_015_as** | **hp:pncA_016_as** | **hp:pncA_017_as** | **hp:pncA_074_His57Asp_rc** | **hp:pncA_018_as** | **hp:pncA_019_as** | **hp:pncA_020_as** | **hp:pncA_021_as** | **hp:pncA_137_rc** | **hp:pncA_022_as** | **hp:pncA_142_rc** | **hp:pncA_024_as** | **hp:pncA_025_as** | **hp:pncA_077_Ser74AGC_rc** | **hp:pncA_078_Ser74AGT_rc** | **SNP M. canettii** | **SNP M. bovis** | **SNP Delhi/CAS** | **SNP silent 74** |
| 4711/09 | --- | --- | genomic DNA |  |  |  |  |  |  |  |  |  |  |  |  |  |  |  |  |  | wt | wt | wt | wt |
|  |  |  | crude culture material |  |  |  |  |  |  |  |  |  |  |  |  |  |  |  |  |  | wt | wt | wt | wt |
| 10298/09 | 2 | His51Asp | genomic DNA |  | x | x | x |  |  |  |  |  |  |  |  |  |  |  |  |  | wt | wt | wt | wt |
|  |  |  | crude culture material |  | x | x | x |  |  |  |  |  |  |  |  |  |  |  |  |  | wt | wt | wt | wt |
| 11237/09 | 3 | Del125-129 | genomic DNA |  |  |  |  |  |  |  |  |  |  |  |  |  |  |  |  |  | wt | wt | wt | wt |
|  |  |  | crude culture material |  |  |  |  |  |  |  |  |  |  |  |  |  |  |  |  |  | wt | wt | wt | wt |
| 5114/09 | 3 | Asp136Ala | genomic DNA |  |  |  |  |  |  |  |  |  |  |  |  |  |  |  |  |  | wt | wt | wt | wt |
|  |  |  | crude culture material |  |  |  |  |  |  |  |  |  |  |  |  |  |  |  |  |  | wt | wt | wt | wt |
| 5100/09 | 3 | Leu151Ser | genomic DNA |  |  |  |  |  |  |  |  |  |  |  |  |  |  |  |  |  | wt | wt | wt | wt |
|  |  |  | crude culture material |  |  |  |  |  |  |  |  |  |  |  |  |  |  |  |  |  | wt | wt | wt | wt |
| 5772/09 | 3 | Arg154Gly | genomic DNA |  |  |  |  |  |  |  |  |  |  |  |  |  |  |  |  |  | wt | wt | wt | wt |
|  |  |  | crude culture material |  |  |  |  |  |  |  |  |  |  |  |  |  |  |  |  |  | wt | wt | wt | wt |

| **Isolate** | **Affected Amplicon** | **Mutation** | **Sample type** | **Hybridization probes (hp) *pncA* A3** | | | | | | | | | | | | | | | | | | | | |
| --- | --- | --- | --- | --- | --- | --- | --- | --- | --- | --- | --- | --- | --- | --- | --- | --- | --- | --- | --- | --- | --- | --- | --- | --- |
|  |  |  |  | **hp:pncA_176_rc** | **hp:pncA_037_rc** | **hp:pncA_179_rc** | **hp:pncA_039_rc** | **hp:pncA_040_rc** | **hp:pncA_186_rc** | **hp:pncA_187_rc** | **hp:pncA_190_rc** | **hp:pncA_191_rc** | **hp:pncA_194_rc** | **hp:pncA_046_rc** | **hp:pncA_198_rc** | **hp:pncA_199_rc** | **hp:pncA_049_rc** | **hp:pncA_253_rc** | **hp:pncA_206_rc** | **hp:pncA_255_rc** | **hp:pncA_053_rc** | **hp:pncA_054_rc** | **hp:pncA_244_rc** | **hp:pncA_258_rc** |
| 4711/09 | --- | --- | genomic DNA |  |  |  |  |  |  |  |  |  |  |  |  |  |  |  |  |  |  |  |  |  |
|  |  |  | crude culture material |  |  |  |  |  |  |  |  |  |  |  |  |  |  |  |  |  |  |  |  |  |
| 10298/09 | 2 | His51Asp | genomic DNA |  |  |  |  |  |  |  |  |  |  |  |  |  |  |  |  |  |  |  |  |  |
|  |  |  | crude culture material |  |  |  |  |  |  |  |  |  |  |  |  |  |  |  |  |  |  |  |  |  |
| 11237/09 | 3 | Del125-129 | genomic DNA |  |  | x | x | x | x | x |  |  |  |  |  |  |  |  |  |  |  |  |  |  |
|  |  |  | crude culture material |  |  | x | x | x | x | x |  |  |  |  |  |  |  |  |  |  |  |  |  |  |
| 5114/09 | 3 | Asp136Ala | genomic DNA |  |  |  |  |  |  | x | x | x | x |  |  |  |  |  |  |  |  |  |  |  |
|  |  |  | crude culture material |  |  |  |  |  |  | x | x | x | x |  |  |  |  |  |  |  |  |  |  |  |
| 5100/09 | 3 | Leu151Ser | genomic DNA |  |  |  |  |  |  |  |  |  |  |  |  |  | x | x | x |  |  |  |  |  |
|  |  |  | crude culture material |  |  |  |  |  |  |  |  |  |  |  |  |  | x | x | x |  |  |  |  |  |
| 5772/09 | 3 | Arg154Gly | genomic DNA |  |  |  |  |  |  |  |  |  |  |  |  |  |  | x | x | x |  |  |  |  |
|  |  |  | crude culture material |  |  |  |  |  |  |  |  |  |  |  |  |  |  | x | x | x |  |  |  |  |

|  |  | Automated detection of the outlier |
| --- | --- | --- |
|  |  | Wrong detection |

The table shows the results of different *pncA* isolates (genomic DNA as well as crude culture material) tested for each amplicon (*pncA* A1, *pncA* A2 and *pncA* A3). The expected theoretical hybridization result based on the *pncA* genotype sequence is indicated by a cross for each of the array probe. The reaction pattern of *pncA* isolates with the probes are marked as green colored boxes if mutations were detected automatically as an outlier by the defined algorithm. A purple colored box represent probes which were determined wrongly as outliers. The four mutant probes within the amplicon *pncA* A2 are shown as slanting lines and the respective results are given in the last four columns (wt – wild type or mut – mutation).
