# Supplementary material for: Rapid microarray-based assay for detection of pyrazinamide resistant Mycobacterium tuberculosis
Source: Diagn Microbiol Infect Dis. 2019 Jun;94(2):147–54. doi: 10.1016/j.diagmicrobio.2018.12.011 (PMC6531379; doi:10.1016/j.diagmicrobio.2018.12.011)
Supplement: Supplementary file 5 — Supplementary material [file mmc5.docx]

## Efficiency of target amplification using the 7500 Real-Time PCR System

Real-Time PCR was performed with fluorescence-labeled TaqMan® probes and primers specific for the *M. tuberculosis* target *pncA*. Due to the length of the *pncA* gene the amplification region was divided into three parts (*pncA* A1, *pncA* A2, and *pncA* A3). An adaption of the PCR buffer was necessary due to the high GC content of *M. tuberculosis* (Cole et al., 1998). For that reason betaine and tetramethylammonium chloride were added to the PCR buffer which are described as potent agents to increase the specificity and the reaction yield of a PCR reaction (Henke et al., 1997; Hung et al., 1990). The target amplification was performed in a 100 µL reaction volume with TET (Tris, ETDA, Tween® 20), 75 mM Tris-HCl (pH 8.5, Alere Technologies GmbH, Jena, Germany), 3 mM magnesium chloride (Sigma-Aldrich, St. Louis, USA), 1 betaine tablet (resulting in 2 M betaine, University Friedrich-Alexander, Erlangen, Germany), 100 mM tetramethylammonium chloride (Alere Technologies GmbH, Jena, Germany), 0.2 mM of deoxynucleoside triphosphates (Thermo Fisher Scientific, Waltham, USA), 0.6 µM of each Cy5 labeled forward primer (Table S2), 0.2 µM of each reverse primer (Table S2), 0.2 µM of corresponding TaqMan® probes (Table S2), 12.5 U of BTR hotstart *taq* (Biotech rabbit, Henningsdorf, Germany) and 10^2^ to 10^4^ copies per reaction of genomic DNA from *M. tuberculosis* H37Rv reference strain (tebu-bio, Offenbach, Germany) and 10^4^ copies per reaction of the internal process control (Eurofins Genomics GmbH, Ebersberg, Germany). Negative assay controls were run using the same PCR reaction mix without any template. The amplification was performed using the 7500 Real-Time PCR system (Applied Biosystems, Foster City, USA) with the subsequent conditions: 95°C for 2 min following 40 cycles at 95°C for 10 s, 64°C for 30 s and 72°C for 30 s. Based on the measured fluorescence values, a standard calibration curve was determined for the target regions *pncA* A1*, pncA* A2 and *pncA* A3. The respective amplification efficiencies were calculated, subsequently.
